# Clinical application of regional cerebral oxygen saturation with disturbance coefficient in children with brain functional injury

**DOI:** 10.3389/fped.2026.1789034

**Published:** 2026-03-25

**Authors:** Shuai Liu, Lihong Hu, Meixian Xu, Xin Zhao, Zexi Wang

**Affiliations:** 1 Department of Intensive Care Medicine, Hebei Children’s Health and Disease Clinical Medical Research Center, Hebei Children’s Hospital, Hebei Medical Key Discipline, Shijiazhuang, Hebei, China; 2 Department of Neurorehabilitation, Hebei Children’s Health and Disease Clinical Medical Research Center, Hebei Children’s Hospital, Shijiazhuang, Hebei, China

**Keywords:** cerebral edema, childhood brain injury, disturbance coefficient, monitoring of cerebral oxygen saturation, non invasive monitoring

## Abstract

**Objective:**

To explore the clinical value of monitoring cerebral oxygen saturation and disturbance coefficient in children with brain injury.

**Methods:**

This study enrolled 92 children with brain dysfunction admitted to the Intensive Care Medicine Department of Hebei Children's Hospital from March 2024 to May 2025 as the research group. They were divided into three subgroups based on age: 1–3, 3–6, and 6–16 years old. During the same period, 150 children who underwent outpatient health examinations were selected as the control group. General information (age, gender, etc.) was collected for both groups. Within 24 h of admission, the research group monitored and recorded DC and rSO_2_ using non-invasive brain edema and cerebral oxygen saturation monitors, while the control group was monitored DC and rSO_2_ during outpatient visits. This study aims to compare different DC and rSO₂ groups, examine their correlation with GCS scores, and evaluate the utility of DC and rSO₂ in assessing clinical status and predicting prognosis in children with brain injury.

**Results:**

There was no statistically significant difference in age and gender between the two groups of patients (*P* > 0.05). The DC and rSO_2_ levels in the research group were lower than those in the control group, and the difference was statistically significant (*P* < 0.05). There was no statistically significant difference (*P* > 0.05) in DC and rSO_2_ levels among children of different genders and etiologies in the research group. There is a positive correlation between DC, rSO_2_, and GCS scores in the research group (rDC = 0.540, *P* < 0.01; rrSO_2_ = 0.509, *P* < 0.01), indicating that the more severe the brain injury, the lower the DC and rSO_2_ levels. In the 3–6 year old group of children, DC may be useful in identifying children at risk for cerebral edema, with an area under the curve of 0.834 (95% CI 0.695–0.973, *P* = 0.001). The optimal cutoff value for DC is 75, with a sensitivity of 75% and a specificity of 78.6%. rSO_2_ was associated with poor clinical outcomes, with an area under the curve of 0.765 (95% CI 0.630–0.899, *P* = 0.003), an optimal cut-off point of 73.5%, a sensitivity of 70.7%, and a specificity of 83.3%.

**Conclusion:**

DC and rSO_2_ may provide complementary information in the evaluation of the condition and prognosis of children with brain injury, as they are non-invasive, dynamic, and have a wider range of clinical applications.

## 1 Introduction

Childhood brain injury, including traumatic brain injury, ischemic hypoxic encephalopathy, cerebrovascular accidents, infectious factors, and perioperative brain injury, is one of the leading causes of childhood death and long-term neurological sequelae in children (1, 2). Due to the rapid development stage of children's brains, their automatic regulation of cerebral blood flow is not yet mature and more susceptible to damage, making the pathological and physiological processes of childhood brain injury more complex and specific (3). Therefore, finding monitoring methods that can early, accurately, and non invasively evaluate the status of cerebral blood flow autoregulation function and brain tissue oxygenation is of great value in guiding clinical treatment and improving the prognosis of pediatric patients. Traditional neurological function monitoring and imaging examinations, such as computed tomography (CT) and magnetic resonance imaging (MRI), have limitations in dynamic and continuous evaluation (4, 5). The non-invasive brain edema dynamic monitor reflects the intracranial condition of patients based on the principles of bioelectrical impedance tomography (BEIT) and electromagnetic disturbance, with disturbance coefficient (DC) as the main output parameter, directly reflecting the dynamic changes in intracranial conditions. Near infrared spectroscopy (NIRS) technology can reflect the local cerebral oxygen saturation (rSO_2_) of brain tissue. Due to its non-invasive, continuous, and bedside real-time monitoring advantages, it has been widely used in clinical monitoring of cerebral oxygen saturation (6, 7). Combining the disturbance coefficient with rSO₂ monitoring, which directly reflects the oxygenation status of brain tissue, is expected to provide a more comprehensive “functional metabolic” monitoring window for the clinical management of pediatric brain injury. This article aims to explore the clinical value of the combined application of the two in various types of brain injuries in children, in order to provide new ideas and basis for improving the precise monitoring and clinical outcomes of childhood brain injuries. Given the heterogeneity of causes of childhood brain injury, this study mainly adopts exploratory methods. The results of this study should be viewed as generating hypotheses about the potential utility of multimodal monitoring strategies, which need to be further validated in larger, pathogen specific cohorts.

## 2 Subjects and methods

### 2.1 Research object

This study included a total of 92 children with brain function impairment admitted to the Intensive Care Medicine Department of Hebei Children's Hospital from March 2024 to May 2025 as the research group. General information of all children was collected and recorded, including gender, age, weight, early warning score, GCS score at admission DC, rSO_2_, and head imaging examination, etc. 150 pediatric patients who underwent outpatient health check ups were selected as the control group during the same period. According to the characteristics of different age groups of children, they are pre stratified into three groups (1–3, 3–6, and 6–16 years old). These age groups correspond to different stages of brain development and maturation of cerebral vascular autoregulation in children. This study was approved by the Medical Research Ethics Committee of Hebei Children's Hospital (ethics review number: 202407-88). All family members of the affected child have signed informed consent forms.

#### 2.1.1 Inclusion and exclusion criteria for the research group

(1) Age between 1 and 16 years old, gender not limited; (2) Acute brain injury confirmed by clinical evaluation and neuroimaging, including but not limited to: traumatic brain injury; Hypoxic-ischemic encephalopathy; Central nervous system infection; Septicemic encephalopathy; Moderate stroke; (3) The skull is intact, and there is no incision or skin defect at the electrode attachment site, but it does not affect the adhesion of the electrode pad; (4) Complete vital signs, laboratory tests, and imaging data can be obtained; (5) The family members of the sick child sign an informed consent form.

##### 2.1.1.1 Exclusion criteria

(1) Skull defect or severe scalp injury affecting data collection; (2) There are factors that affect the accuracy of cerebral oxygen monitoring, such as severe anemia, hyperbilirubinemia, etc.; (3) Severe congenital heart disease or circulatory failure, active intracranial hemorrhage, or uncontrolled status epilepticus; (4) Previous conditions include delayed neurological development, cerebral palsy, genetic metabolic disorders, etc.; (5) Expected survival time <72 h or plan to abandon treatment.

#### 2.1.2 Inclusion and exclusion criteria for control group

(1) Age between 1 and 16 years old, gender not limited; (2) Normal growth and development; (3) No recent history of fever, infection, or traumatic brain injury; (4) No history of epilepsy, convulsions, encephalitis, syncope, genetic metabolic diseases, or cranial diseases (intracranial space occupying lesions, hydrocephalus, etc.); (5) The surface of the skull skin is intact.

##### 2.1.2.1 Exclusion criteria

(1) Children who cannot cooperate in completing the collection; (2) Individuals with severe allergies to electrode pads; (3) Local skin damage affects electrode pad adhesion.

### 2.2 Research methods

This study is a prospective observational study and does not involve any intervention measures. All children who participated in this study received the same treatment as those who did not participate, such as anti infection, intracranial pressure reduction, respiratory support, fluid replacement for anti shock, analgesia and sedation, and brain function assessment.

#### 2.2.1 Monitoring method

All children are in a calm state during brain function monitoring, with a Comfort-B score between 11 and 22 points. Their body temperature, heart rate, blood pressure, and breathing are all within normal ranges. All patients should undergo cerebral edema and cerebral oxygen monitoring within 24 h of admission.

##### 2.2.1.1 DC monitoring

All enrolled children were monitored using BORN-BE-IVA non-invasive brain edema monitor (Chongqing Boenfuk Medical Equipment Co., Ltd.). Using electrical impedance tomography technology and electromagnetic disturbance principle to monitor intracranial conditions. It explains the changes in internal conductivity of the body by applying a specific current to the tissue and measuring the voltage between electrodes located at specific positions on the body surface when a specific amount of current flows through the body (8), with DC being its main monitoring parameter. By monitoring DCs, quantitative data on brain edema can be provided to clinical physicians. For measurement, the nurse removes the hair above the temporal area of the patient's ear screen to ensure a smooth scalp in the spare skin area at the wing point. Connect the electrode pads by a physician:Use alcohol or disinfectant wipes to degrease and disinfect the electrode bonding area 2–3 times. Connect the lead wires to the device and fasten the electrode pads in brown, green, white, and black order. The four electrode pads are symmetrical on both sides, and the center (button) of the rear electrode pad is aligned with the highest point of the auricle above the external auditory canal. The lower edge of the electrode pad overlaps with the extension line of the outer corner of the eye. The front electrode pad is closely attached to the rear electrode pad and placed side by side, using the “meter” method for bonding. The measurement time is 30 min each time, recorded every 2 s, and the average value of the most stable segment observed during the measurement process for 15 min is the measurement DC.

##### 2.2.1.2 rSO₂ monitoring

rSO_2_ was measured using a non-invasive brain oxygen monitor (BeneVision N12). The measurement principle is to place the sensor probe on the forehead of the child. The near-infrared light emitted by the light-emitting diode enters the forehead tissue, undergoes emission, scattering, and absorption, forming an arc-shaped path. Finally, it is detected by the surface photodetector and deep photodetector on the forehead sensor. The absolute oxygen content, tissue hypoxia, and total hemoglobin content are evaluated by measuring the different times a single photon passes through the susceptible tissue, and the local cerebral oxygen saturation is obtained (9, 10). For measurement, the nurse cleans the forehead skin of the child, removes hair, oil, or dirt, and attaches the sensor to the left and right foreheads. The probes on both sides are located about 1 cm from the lower edge of the eyebrow arch and 0.5–1 cm from the midline of the forehead, while avoiding hair and eyebrows as much as possible. Press the start button, and the instrument will automatically collect data. When the signal strength reaches a stable state, record the rSO_2_ value displayed on the screen.

### 2.3 Observation indicators

Collect general information of all children, including gender, age, warning score, blood biochemical indicators, blood gas analysis, mechanical ventilation time, DC at admission, rSO_2_, Glasgow Coma Scale (GCS) score head imaging examination, length of hospital stay, etc. Collect general information of the control group of children during the same period, including age, gender, DC, rSO_2_.

### 2.4 Statistical methods

SPSS 22.0 statistical software was used to perform statistical processing on the data. Measurement data that conforms to normal distribution are represented by mean ± standard deviation (*x̅* ± *s*), while measurement data that does not conform to normal distribution are represented by median (interquartile range). Comparisons between two or more groups of metric data that conform to normal distribution are conducted using *t*-test and analysis of variance, while comparisons between groups of continuous variables that do not conform to normal distribution are conducted using Mann Whitney *U* test; The comparison of count data between groups was conducted using the chi square test or Fisher's exact test. Conduct correlation studies using Pearson correlation analysis (normal distribution data) and Spearman correlation analysis (non normal distribution data). Draw receiver operating characteristic (ROC) curves for the subjects, calculate the area under the curve (AUC), critical value, and determine its specificity and sensitivity. *P* < 0.05 is considered statistically significant.

## 3 Results

### 3.1 Comparison of general information between two groups of children

This study included a total of 92 children with brain injury, including 56 boys and 36 girls, with a median age of 5.0 (3.0–7.0) years. The control group consisted of 150 cases, including 70 boys and 80 girls, with a median age of 5.5 (3.0–7.0) years. There was no statistically significant difference in age and gender between the two groups of patients (*P* > 0.05). The DC and rSO_2_ levels of the study group were lower than those of the control group, and the difference was statistically significant (*P* < 0.05), as shown in Table 1. There was no statistically significant difference (*P* > 0.05) in DC and rSO_2_ levels among children of different genders and etiologies in the research group, as shown in Table 2. However, due to the small sample size of certain subgroups (such as stroke, *n* = 4), these findings should be interpreted with caution and considered as exploratory results.

**Table 1:** General information comparison of two groups of children.

Age group	Indicator	Research group	Control group	*t*/*x*^2^	*P*
1–3 years old	Gender (Example)			1.873	0.171
	Male	16	17		
	Female	12	25		
	Age (*x̅* ± *s*, years)	2. 12 ± 0.79	2. 17 ± 0.79	0.247	0.806
	DC	65. 11 ± 5.57	80.93 ± 6.26	10.814	0.000
	rSO_2_ (%)	70.04 ± 6.62	75.74 ± 9.94	2.663	0.010
3–6 years old	Gender (Example)			2.532	0.112
	Male	22	25		
	Female	11	26		
	Age (*x̅* ± *s*, years)	4.94 ± 0.79	5.00 ± 0.85	0.329	0.743
	DC	75.67 ± 6.30	89.92 ± 5.56	10.894	0.000
	rSO_2_ (%)	69.67 ± 7.08	74.31 ± 10.86	2.175	0.033
6–16 years old	Gender (Example)			0.644	0.422
	Male	18	28		
	Female	13	29		
	Age (*x̅* ± *s*, years)	9.55 ± 2.77	8.72 ± 2.52	1.424	0.158
	DC	87.81 ± 8.65	99.98 ± 8.69	6.289	0.000
	rSO_2_ (%)	73.29 ± 8.51	74.33 ± 9.23	2.064	0.042

**Table 2:** Comparison of DC and rSO_2_ between different subgroups of children in the study group.

Indicator		Number of cases (examples)	DC	Test value	*P*	rSO_2_ (%)	Test value	*P*
Age	1–3 years old	28	65. 11 ± 5.57	77.972^a^	0.000	74.57 ± 7.79	0.118^a^	0.889
	3–6 years old	33	75.67 ± 6.30			72.97 ± 10.47		
	6–16 years old	31	87.81 ± 8.65			73.29 ± 8.51		
Gender	Male	56	75.86 ± 10.10	0.714^b^	0.477	77.34 ± 9.51	1.378^b^	0.172
	Female	36	77.61 ± 12.25			74.50 ± 9.86		
Etiology	Intracranial infection	31	76.23 ± 11.79			75.61 ± 9.74		
	Hypoxic-ischemic encephalopathy	22	76.68 ± 12.45	0.285^a^	0.887	75.91 ± 10.53	0.264^a^	0.900
	Traumatic brain injury	9	76.78 ± 8.35			74.44 ± 9.55		
	Sepsis-associated encephalopathy	26	77.58 ± 11.91			77.69 ± 9.56		
	Stroke	4	71.00 ± 9.63			77.25 ± 9.32		
Head imaging	Signs of cerebral edema (+)	38	73.34 ± 9.31	2.063^c^	0.042	72.47 ± 10.30	3.237^c^	0.002
	Signs of cerebral edema (−)	54	78.08 ± 11.73			78.85 ± 8.45		
GCS score	<9 points	41	72.37 ± 11.20	2.081^c^	0.040	73.78 ± 9.98	2.217^c^	0.029
	≥9 points	51	77. 10 ± 8.20			78.20 ± 9.08		
Prognosis	Death	10	68.40 ± 8.24	2.012^c^	0.047	68.50 ± 8.86	2.766^c^	0.007
	Survive	82	75.95 ± 11.49			77. 17 ± 9.41		

^a^ Represents analysis of variance.

^b^ Represents chi square test.

^c^ Represents *t*-test.

### 3.2 DC, rSO_2_ for disease assessment

**Figure 1:**
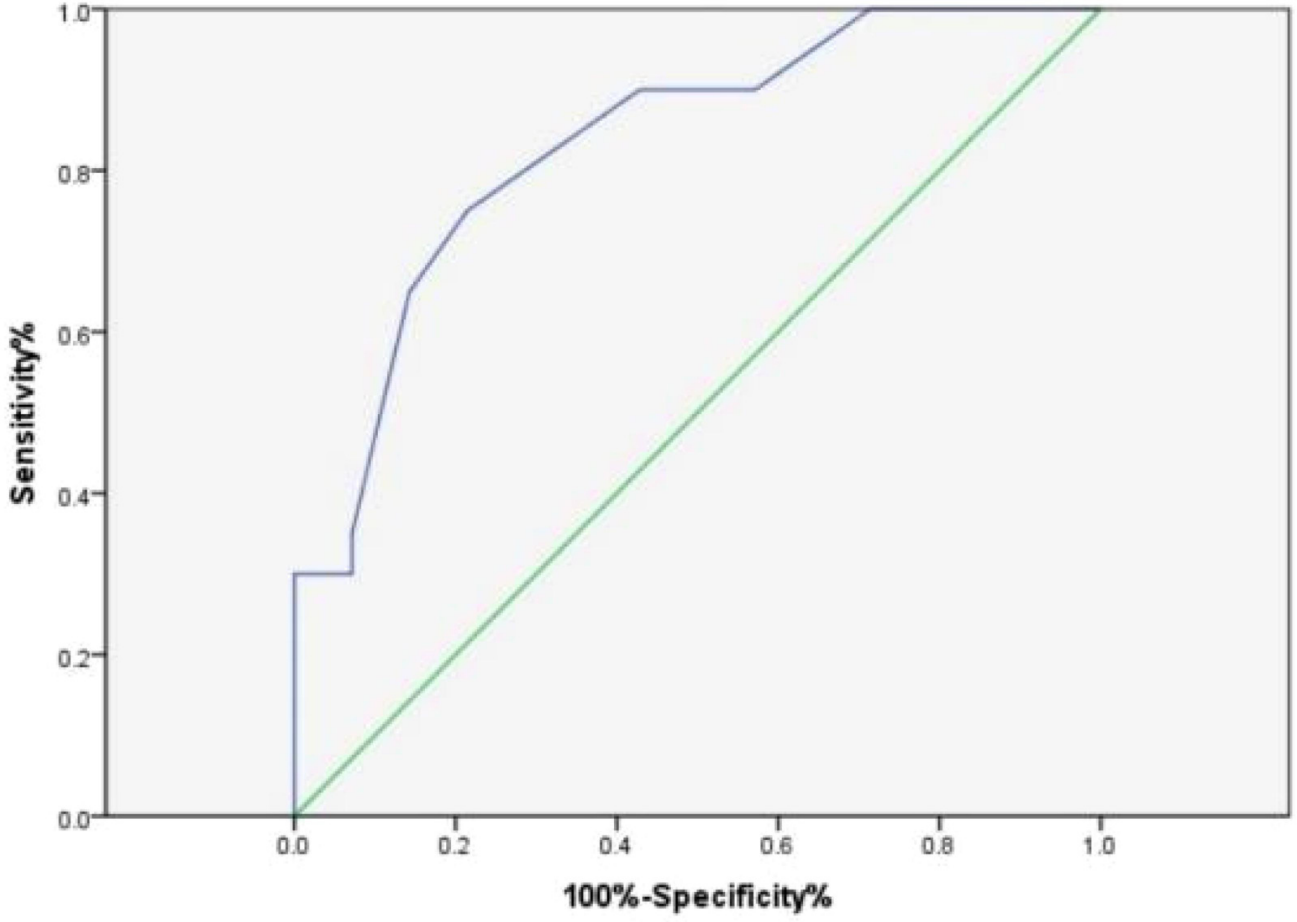
ROC curve of DC prediction for cerebral edema in 3–6 year old children.

**Figure 2:**
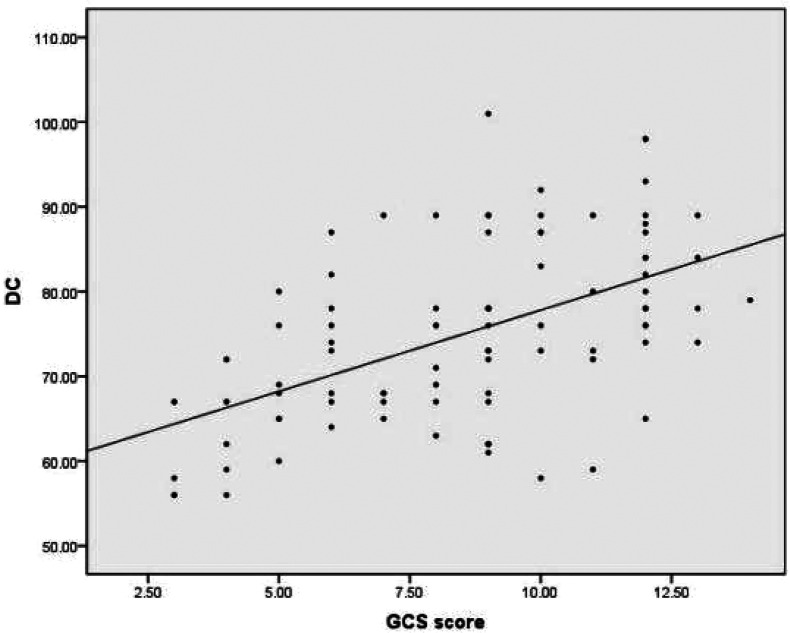
Correlation between DC and GCS scores.

**Figure 3:**
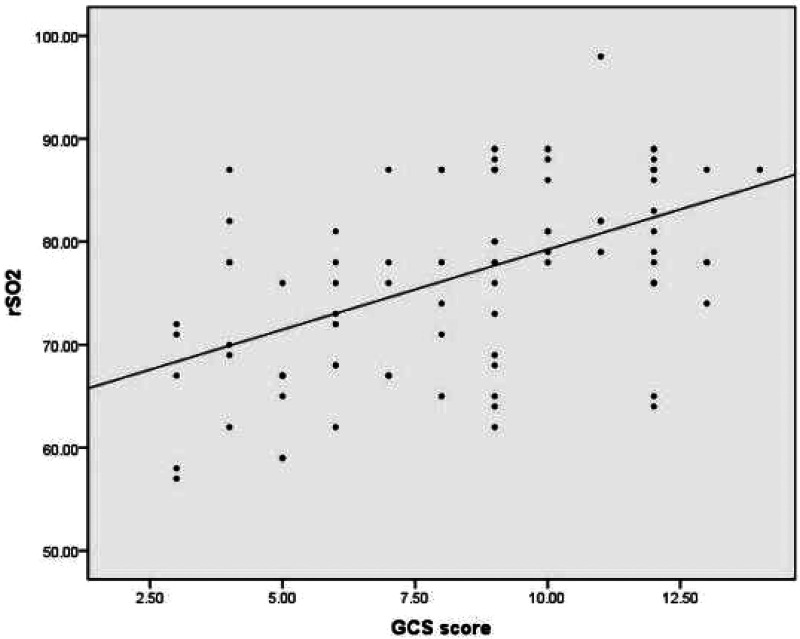
Correlation between rSO_2_ and GCS score.

DC and rSO_2_ were significantly lower in children with severe brain injury (GCS score < 9) compared to those with mild to moderate brain injury (GCS score ≥ 9) in the disease assessment study group, and the difference was statistically significant (*P* < 0.05). Head imaging showed a significant decrease in DC and rSO_2_ levels in children with cerebral edema, and the difference was statistically significant (*P* < 0.05) compared to children without cerebral edema, as shown in Table 2. ROC curve analysis revealed a significant association between DC and brain edema in the 3–6 year old children group in subgroup analysis (AUC = 0.834, 95% CI: 0.695–0.973, *P* = 0.001), with an optimal cutoff value of 75 (sensitivity 75%, specificity 78.6%), as shown in Figure 1. No statistical association was observed in other age groups (1–3 and 6–16 years), which may be attributed to smaller subgroup sample sizes or age-related physiological differences. The correlation analysis suggests that there is a positive correlation between DC, rSO_2_, and GCS scores in the study group (rDC = 0.540, *P* < 0.01; rrSO_2_ = 0.509, *P* < 0.01), indicating that the more severe the brain injury, the lower the DC and rSO_2_ levels, as shown in Figures 2, 3.

### 3.3 DC and rSO_2_ for prognostic evaluation

**Figure 4:**
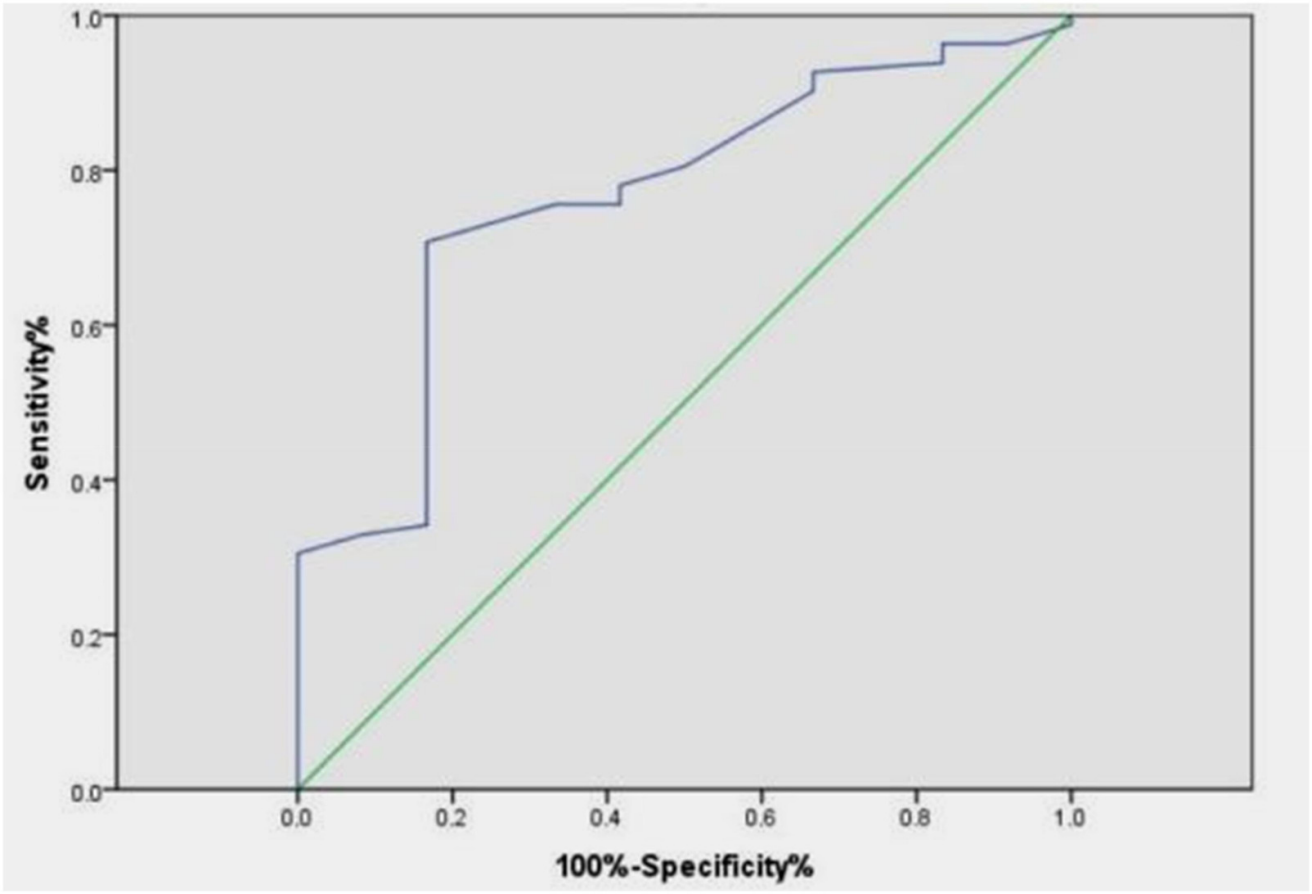
ROC curve of rSO_2_ predicting poor prognosis.

The DC and rSO_2_ levels in the death group were significantly lower than those in the survival group, and the difference was statistically significant (*P* < 0.05), as shown in Table 2. Through ROC curve analysis, it was found that rSO_2_ has a good predictive ability for poor prognosis, with an area under the curve of 0.765 (95% CI 0.630–0.899, *P* = 0.003), an optimal cutoff point of 73.5%, sensitivity of 70.7%, and specificity of 83.3%, as shown in Figure 4.

## 4 Discussion

Childhood brain injury is one of the leading causes of child mortality and long-term neurological dysfunction worldwide (11). Its etiology is complex, including traumatic brain injury, hypoxic-ischemic encephalopathy, stroke, intracranial infection, status epilepticus, etc. The injury not only causes acute neurological deficits, but also may affect subsequent brain development, cognitive function, and behavioral shaping, bringing a heavy burden to families and society (12). Therefore, early intracranial monitoring and evaluation play a crucial role in improving prognosis and enhancing the quality of life of critically ill children.

The traditional evaluation and monitoring methods mainly rely on cranial imaging examinations, which cannot be continuously, dynamically, and bedside monitored. These technologies are costly for critically ill children and there is a risk ofunexpected events during patient transportation. New monitoring technologies based on principles of optics, audiology, ophthalmology, and acoustics have been explored, but their clinical value needs to be fully evaluated (13). In recent years, non-invasive brain edema monitoring devices have been widely used in clinical practice, and their bedside, non-invasive, and dynamic real-time capabilities have been recognized by clinical physicians. Normal brain tissue has stable electrical impedance characteristics; When pathological processes such as brain tissue edema or bleeding occur, the electromagnetic field will be altered. These changes are detected by surface electrodes and converted into quantitative DC values, providing real-time information on brain edema (14, 15). There is a certain relationship between DC and cerebral edema, which is closely related to the automatic regulation function of cerebral blood flow: when this function is impaired, the static water pressure of cerebral capillaries increases, which may lead to the formation of vascular edema, an increase in extracellular fluid, and changes in local brain tissue impedance characteristics, resulting in a decrease in DC value. Therefore, the DC value can serve as an indirect indicator of automatic regulation function, and its decrease may indicate the loss of automatic regulation ability, thereby increasing the risk of secondary brain injury. The monitoring of cerebral oxygen saturation based on near-infrared spectroscopy technology hasbecome an indispensable functional monitoring tool for intensive care. The measurement principle is to place the sensor probe on the forehead of the child, and the near-infrared light emitted by the light-emitting diode enters the forehead tissue, causing emission, scattering, and absorption, forming an arc-shaped path. Finally, it is detected by the surface photodetector and deep photodetector on the forehead sensor. The absolute oxygen content, tissue hypoxia, and total hemoglobin content are evaluated by measuring the different times a single photon passes through the susceptible tissue, and the local cerebral oxygen saturation is obtained (16, 17). It evaluates the oxygenation status of local brain tissue in a non-invasive and continuous manner, providing a unique window for real-time understanding of brain oxygen supply and demand balance and early warning of cerebral ischemia and hypoxia events. It has the characteristics of simple operation, sensitive response, rapid, and real-time monitoring (16). Monitoring DC and rSO_2_ can provide real-time, bedside, and multidimensional evaluation of pediatric brain injury through two interrelated core pathophysiological processes: brain tissue oxygen metabolism and brain edema evolution.

This study found that the DC of the children in the study group was lower than that of the control group. Head imaging showed a significant decrease in DC in children with cerebral edema, and the difference was statistically significant compared to children without cerebral edema. This suggests that children with brain injury have varying degrees of cerebral edema, which can be quantitatively reflected by DC. ROC curve analysis shows that DC has certain diagnostic value for cerebral edema. In children aged 3–6 years, DC ≤ 75 indicates the presence of cerebral edema, which requires special attention in clinical practice. This is consistent with current research confirming that DC is associated with intracranial pressure and brain edema in adult patients. However, the diagnostic efficacy of DC for cerebral edema only showed good value in the 3–6 year old group, which may be related to the inherent differences caused by age stratification. Firstly, the age range of 3–6 years old may represent a developmental window period, during which cranial calcification is basically completed, intracranial structures are relatively stable, and DC can more accurately capture changes in electrical characteristics caused by edema. Secondly, the youngest age group (1–3 years old) has thinner skulls, higher brain tissue water content, lower and more fluctuating DC values, resulting in a decreased sensitivity of DC to pathological edema, and a relatively small sample size (*n* = 28) included. Thirdly, for older children (6–16 years old), thickening of the skull may weaken the bioimpedance signal, potentially reducing the sensitivity of DC detection for brain edema. Therefore, it is not accidental that DC exhibits the best diagnostic value in children aged 3–6 years old. It reflects that children in this age group are in a relatively stable period of brain structural development, and DC can more specifically reflect changes in brain edema. In the future, it is necessary to conduct larger and more comprehensive studies using age stratification design to establish reference ranges and diagnostic thresholds for specific ages. The GCS score is the cornerstone of clinical evaluation, treatment decision-making, and prognosis assessment for acute brain injury. This study found a positive correlation between DC, rSO_2_, and GCS score. The more severe the coma, the lower the DC and rSO_2_. The rSO_2_ of the deceased group was significantly lower than that of the surviving group. ROC curves showed that when rSO_2_ was below 73.5%, it could be used as a prognostic indicator for poor prognosis. Vilk et al. (17) found that rSO_2_ < 68% within 1 h of admission is associated with an increased risk of death. These findings are consistent with observations in the neonatal population. Studies in neonates with hypoxic-ischemic encephalopathy have demonstrated that lower cerebral rSO_2_ values during the first days of life are associated with adverse neurodevelopmental outcomes. Kazanasmaz et al. reported (18) that in asphyxiated neonates treated with therapeutic hypothermia, CrSO_2_ values were significantly lower than in healthy controls (right: 67.38% vs. 80.28%; left: 66.73% vs. 79.14%), with cutoff values of ≤72% (left) and ≤74% (right) showing predictive utility, these findings emphasize that rSO_2_ monitoring may have broad applicability across the entire pediatric age range. However, due to changes in brain metabolism and hemoglobin concentration with age, the optimal threshold may vary by age, highlighting the importance of developing age-specific reference data and providing direction for future research. DC and rSO_2_ not only effectively reflect the real-time pathological and physiological status of children with brain injury, but also have a correlation with the severity of the disease and the prognosis of neurological function. This provides a new and valuable tool combination for accurate monitoring and prognosis evaluation of children with brain injury.

As a single center observational study, this study has a relatively limited sample size and may have selection bias. Secondly, the rSO_2_ monitored by NIRS mainly reflects the oxygenation status of surface brain tissue, which has limitations in evaluating deep structures. Thirdly, we acknowledge that there are certain limitations to the selection of the control group. There are significant differences in pathology and clinical outcomes between healthy and critically ill children in outpatient settings, and this controlled design may introduce potential confounding factors. However, this study focuses more on exploring the dynamic changes and prognosis of children with brain injury. Although there are physiological differences between the two groups of children, the role of the control group in this study is mainly to provide a reference value for children of the same age in a normal physiological state. Of course, future research should consider establishing more rigorous control groups, such as including non brain injury ICU patients (such as respiratory failure patients without neurological involvement), in order to more accurately separate the effects of brain injury itself. In addition, this study included children with brain injuries of different etiologies, and the sample size of each subgroup was limited, making it difficult to determine the specific effects of specific etiologies. DC and rSO_2_ may reflect a common ultimate pathophysiological pathway rather than pathogen specific mechanisms. Therefore, further research is needed in larger stratified etiological cohorts in the future to validate these observations and determine whether the optimal monitoring threshold varies depending on the etiology. Finally, although an association with prognosis has been found, the exact causal relationship and optimal intervention threshold still require larger prospective studies to establish.

In summary, this exploratory study suggests that monitoring brain oxygen saturation and disturbance coefficient can provide valuable reference information for the severity and prognosis of childhood brain injury from two key dimensions: brain tissue oxygenation status and cerebral blood flow autoregulation function. These findings suggest the potential application value of multimodal non-invasive monitoring methods in pediatric neurological intensive care, and provide a hypothesis basis for future research. However, due to the limitations of this study, the current research results are not sufficient to support the establishment of a unified clinical intervention threshold. In the future, larger sample, multi center, and etiological stratified prospective studies are needed to establish the optimal intervention threshold and determine the clinical efficacy of different etiologies and age groups.

## Data Availability

The original contributions presented in the study are included in the article/Supplementary Material, further inquiries can be directed to the corresponding author.
